# Pagnes et prévention en santé

**DOI:** 10.48327/mtsi.v4i4.2024.578

**Published:** 2024-10-22

**Authors:** Jean-Loup REY, François DENIAUD

**Affiliations:** Groupe d'intervention en santé publique et épidémiologie (GISpE). 20 rue des Crotes. 04180. Villeneuve, France; Centre médico-social, Ville de Paris, 3 rue de Ridder, 75014, Paris, France

**Keywords:** Pagne, Prévention, Sida, Relations hommes-femmes, Dabou, Abidjan, Côte d’Ivoire, Afrique subsaharienne, Loincloth, Prevention, AIDS, Gender relationships, Dabou, Abidjan, Côte d’Ivoire, Sub-Saharan Africa

## Abstract

Les auteurs rapportent des actions menées dans les années 90 en Côte d’Ivoire utilisant des méthodes originales de prévention du sida. Ces actions sont basées sur l'utilisation des pagnes. Le pagne est un tissu imprimé très prisé en Afrique. Il présente la caractéristique essentielle d’être approprié par les acheteuses pour manifester principalement les questions de relations entre femmes et hommes. Cette appropriation se manifeste par le fait que les pagnes sortent de la fabrique avec un nom commercial et que le plus souvent les utilisatrices les dénomment autrement en rapport avec leurs préoccupations.

Des études sur le terrain ont été réalisées pour recenser les caractéristiques des pagnes les plus vendus sur les marchés d’Abidjan. Par ailleurs, des entretiens et activités ont été réalisés avec des lycéens d’Abidjan et de Dabou (ville à 50 km d’Abidjan) qui ont permis la fabrication d'un pagne dénommé « *entre nous* » qui reprend les logos de prévention du sida et qui a fait l'objet de plusieurs manifestations de sensibilisation. Un autre projet porté par une association féminine de lutte contre le sida et basé sur la modification d'un pagne à la mode, n'a pas pu aboutir.

Le pagne reste un objet très présent dans les sociétés africaines, avec des développements commerciaux et artistiques; il est logique de penser que son utilisation en prévention a de l'avenir.

## Abstract (en)

The authors report on activities carried out in the 1990s in Côte d’Ivoire using original AIDS prevention methods. These initiatives were based on the use of loincloths. The loincloth is a printed fabric that is highly valued in Africa. It has the essential characteristic of being appropriated by women buyers to express issues of relations between women and men. This appropriation can be seen in the fact that loincloths leave the factory with a trade name, but in most cases are given a different name by the users to reflect their own concerns.

Field survey was conducted to identify the characteristics of the most popular loincloths sold in Abidjan markets. In addition, inter-views and activities were conducted with high school students in Abidjan and Dabou (a town 50 km from Abidjan), resulting in the production of an *“entre nous*” *[between us]* loincloth which uses AIDS prevention logos, and has been used in several awareness-raising events. Another project by a women's anti-AIDS association, based on the modification of a fashionable loincloth, was unsuccessful.

The loincloth is still very much a part of African society, with commercial and artistic developments; it's logical to think that its use in prevention has a future.

## Introduction

**Figure 1:**
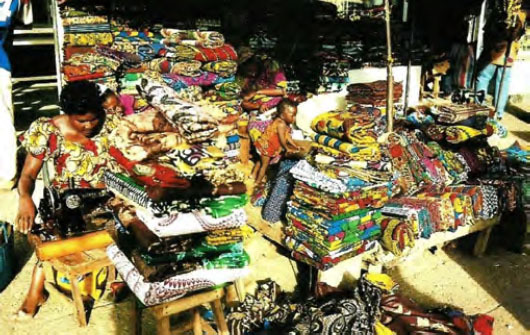
Marché en Côte d’Ivoire (crédit photo : Jean-Loup Rey)

Le pagne africain est vendu sur tous les marchés africains (Fig. 1) et toutes les femmes d’Afrique subsaharienne, même les plus occidentalisées, portent un jour une tenue en pagne. Ces tissus font partie de l'histoire africaine ancienne [6] ou de l'histoire plus récente avec les wax [5].

Dans les années 90, nous avons envisagé d'utiliser cette mode comme moyen de sensibilisation et de prévention du sida. Nos travaux ont pour objet d'exposer les actions menées dans ce but en Côte d’Ivoire, essentiellement à Abidjan, capitale économique. À l’époque, les téléphones portables étaient rares, l'utilisation d'internet aléatoire et peu accessible en raison de son coût.

## Préparation et usage des pagnes

Trois actions ont été menées.

1. En 1993, la sociologue Corinne Ginoux-Pouyaud [4] a interrogé dans 5 marchés de la ville d’Abidjan les acheteuses des 32 pagnes les plus vendus d'après les informations fournies par les marchands. Les couleurs, les motifs de ces pagnes et les noms donnés par les vendeuses et les acheteuses ont été recensés. Ces noms, différents des noms attribués par les fabricants, sont le signe de l'appropriation du pagne par les femmes, et parfois par les hommes.
2. En 1993, François Deniaud [3], médecin sociologue, a mené plusieurs enquêtes et entretiens avec une centaine de lycéens d’Abidjan et de Dabou sur l'utilisation des préservatifs et sur leurs idées pour une prévention adaptée.
3. En 2001, une nouvelle enquête auprès de 50 vendeuses de pagnes d’Abidjan a été réalisée avec le Comité de femmes pour la lutte contre le sida (COFEL).

Dans la première enquête de 1993, les couleurs dominantes des 32 pagnes analysés sont le jaune et le rouge (18 fois) suivies du bleu et du blanc. Les dessins étaient le plus souvent abstraits (volutes, spirales, torsades, grilles, bandes, croix). Dans 25 % des cas, on retrouvait des dessins reprenant des motifs d'anciens pagnes, chevrons, hachures, jets d'encre.

Concernant le nom donné par les femmes, les thèmes généraux identifiés sont les relations femmes/hommes (25 fois), le bonheur (19 fois), la beauté (11 fois), l'argent (10 fois) et la santé (3 fois). Les sujets directement évoqués au sein de ces thèmes sont la jalousie (5 fois), la richesse (5 fois), les relations amoureuses (4 fois), la beauté (4 fois), le couple, la santé, l'ingratitude (2 fois chacun).

**Figure 2:**
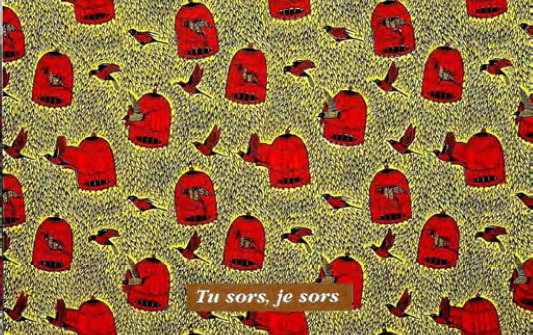
Pagne « *tu sors je sors* » (crédit photo : Maurice Ascani)

**Figure 3:**
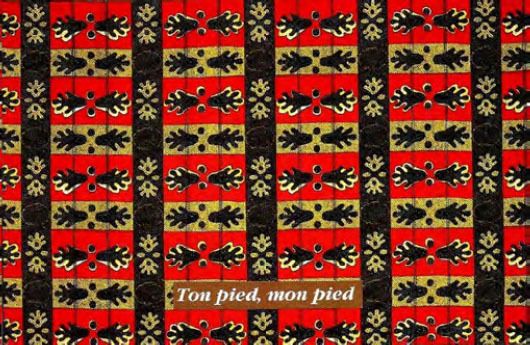
Pagne « *ton pied mon pied* » (crédit photo : Maurice Ascani)

**Figure 4:**
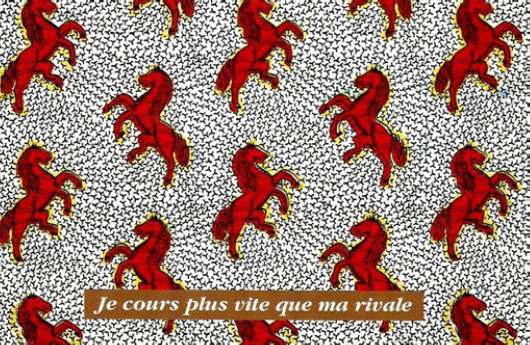
Pagne « *je cours plus vite que ma rivale* » (crédit photo : Maurice Ascani)

**Figure 5:**
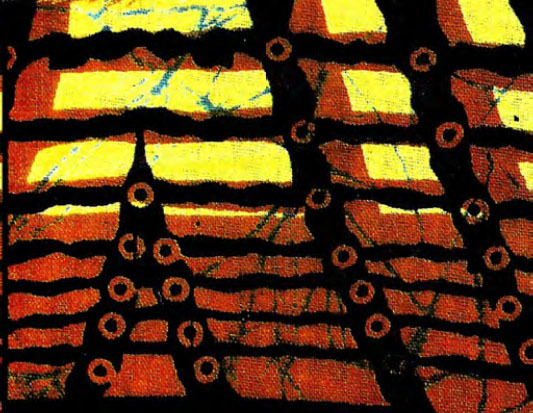
Pagne « *carrefour du bonheur* » (crédit photo : Jan-Loup Rey)

**Figure 6:**
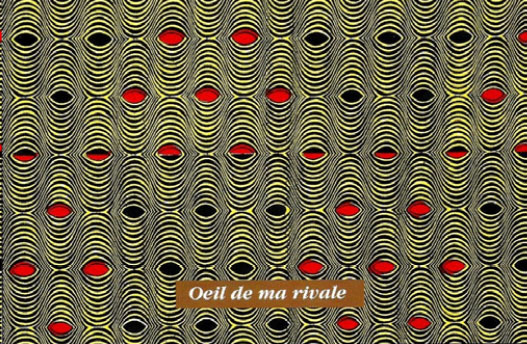
Pagne « *œil de ma rivale* » (crédit photo : Maurice Ascani)

**Figure 7:**
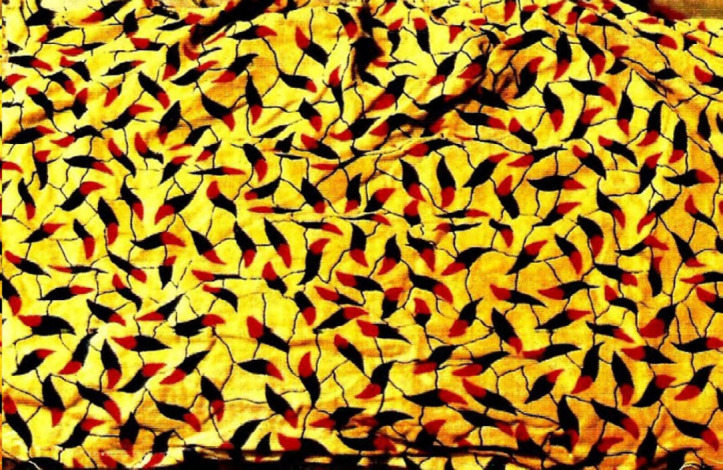
Pagne «SIDA» mis sur le marché en 1987 lors de l'annonce officielle du sida (crédit photo : Jean-Loup Rey)

Certaines images donnent un sens direct au nom comme le pagne avec une cage et deux oiseaux dénommé « *tu sors, je sors* » (Fig. 2), celui (Fig. 3) avec deux pieds sur fond jaune et deux sur fond rouge dénommé « *ton pied, mon pied* » (là où le mari va, la femme va). Un 3^e^ pagne avec deux chevaux, surnommé « *je cours plus vite que ma rivale* » (Fig. 4) est réédité avec les deux précédents depuis plusieurs années. Des pagnes aux motifs géométriques sont appelés « *carrefour du bonheur »* ou « *attends-moi au carrefour* » (Fig. 5). Des motifs plus ou moins organiques suscitent les noms suivants « *œil de ma rivale* » (Fig. 6) ou « *la pluie ne mouille pas le corps d'une belle fille* ». Enfin, il existe les pagnes événementiels, plus connus, imprimés à l'occasion de l'anniversaire de l'indépendance ou du président ou lors de la visite d'un chef d’État étranger. Ils n'ont pas toujours un grand succès. Ce fut le cas d'un pagne - surnommé « sida » - fait de dessins amiboïdes rouges sur fond jaune, sorti en 1987, lors de la reconnaissance officielle de l’épidémie de sida en Côte d’Ivoire par le ministre de la santé (Fig. 7). De même, le pagne imprimé lors du décès du président Houphouët Boigny a été un échec d'après les vendeuses interrogées par les femmes du COFEL.

Certains pagnes existent depuis les années 70 avec parfois des noms différents : « *œil de ma rivale* » s'appelait auparavant « *jalousie* ». Ces rééditions montrent le succès de ces vêtements qui le plus souvent évoquent les problèmes des relations femmes/hommes. En fait, ces pagnes interviennent dans les approches de séduction ou au contraire de rejet, les acheteuses reconnaissant jouer sur l'ambiguïté du port du pagne. Il est, en général, peu admis de draguer ostensiblement un homme (surtout marié) et porter tel ou tel pagne exprimera ce non-dit « *chéri attends-moi au carrefour* », « *carrefour du bonheur* », « *cœur brisé* », mais aussi « *belle dame* », ou un aspect financier : « *femme capable* », « *mari capable* ».

Au total, toute une série de pagnes permet aux femmes d'exposer leurs opinions et leurs revendications vis-à-vis de leur situation dans les rapports femmes/hommes et contre le sexisme : « *j'ai le droit d'aller où tu vas* », « *je mets ce que je veux* », « *l'homme est ingrat* ».

Il nous est donc apparu intéressant d'utiliser le pagne comme moyen de prévention du VIH puisque ce sont des moyens de communication très utilisés et que les messages véhiculés sont très souvent en rapport avec les relations affectives ou sexuelles [4].

**Figure 8:**
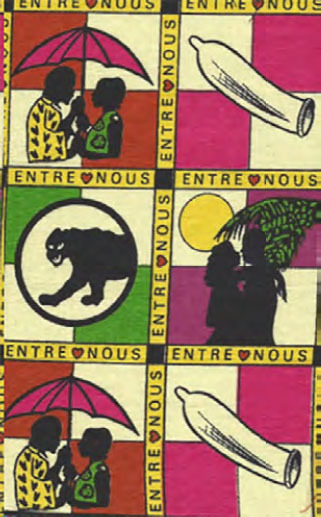
Pagne « *entre nous* » (crédit photo : Jean-Loup Rey)

**Figure 9:**
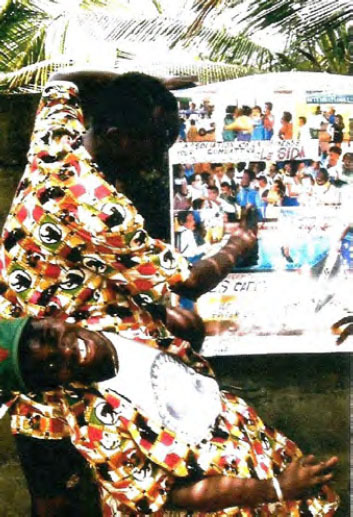
Lancement et diffusion du pagne « *entre nous* » (crédit photo : François Deniaud)

En 1993, un pagne « entre nous » a été réalisé selon les indications des lycéens (Fig. 8). Il reprend quatre dessins en relation avec la prévention du sida. Ce pagne a été créé et diffusé (Fig. 9) par François Deniaud avec les lycéens participants, et financé par le Programme national de lutte contre le sida, l’Office de la recherche scientifique et technique outre-mer (ORSTOM, devenu l’Institut de recherche pour le développement, IRD), le PSI (société de marketing social) et la Mission française de coopération [3]. Ce pagne est entré en 1995 dans le catalogue du matériel d'information et de prévention du CRIPS (Centre régional d'information et de prévention du sida) [1]. D'autres actions d'ethno-prévention ont accompagné la commercialisation de ce pagne : affiche créée par les jeunes, concours de dessins et de slogans, cassette de musique « *Chaussez capote* ».

Une autre piste prévoyait de prendre un pagne existant et prisé comme la cage avec les oiseaux (Fig. 2) « *tu sors, je sors* », en ajoutant un préservatif pour l'oiseau en cage « *tu sors, je me protège* » ou avec un préservatif pour chaque oiseau « *tu sors, je sors, on se protège* ».

En 2001, l'enquête du COFEL a concerné 250 vendeuses rencontrées au hasard sur les marchés, à partir d'un guide d'entretien sur la nature des pagnes, leur commercialisation, les caractéristiques des acheteuses, l'usage prévu et les motivations du choix.

Les principaux résultats montrent que les surnoms des pagnes se rapportent à des faits de société, des personnalités, des évènements publics, traditionnels ou religieux, des situations malheureuses, et à cinq thèmes se rapportant aux relations femmes/hommes :

• Pagnes sur la jalousie : « *œil de ma rivale* », « *maîtresse yako* » (yako voulant dire courage en baoulé), « *ton pied, mon pied* », « *chéri ne me tourne pas le dos* », ou « *si tu sors, je sors* ».

- Pagnes sur la déception : « *mille ans pour rien* », « *si je savais* ».
- Pagnes sur les risques du vagabondage sexuel : « *le monde est gâté* », « *bobodouman* » (nom dioula d'une maladie sexuellement transmissible).
- Pagnes inspirés des séries brésiliennes très prisées à l’époque : « *Marimar* » (rencontre entre Marimar, une jeune fille pauvre, et un jeune homme riche), « *sac à puces* » (nom du chien de Marimar), « *mallette de Ricardo* », « *Rosa* » et « *Matildé* », noms des personnages des séries.
- Pagnes sur la famille : « *fleurs de mariage* », « *famille* », « *gros cœur* », « *l'enfant est mieux que l'argent* ».

Cette étude a confirmé l'assimilation de concepts liés à quelques pratiques rituelles dans l'usage des pagnes [2].

**Figure 10:**
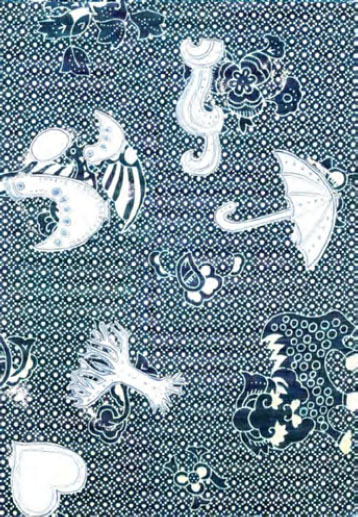
Pagne « *sac à puces* » détourné : proposition association COFEL (crédit photo : Jean-Loup Rey)

Ce travail a débouché sur la proposition de créer un pagne à partir du pagne très prisé « *sac à puces* » (nom venant de la série télévisée brésilienne *Marimar),* en gardant les dessins de base et en y ajoutant des symboles de lutte contre le sida (parapluie, baobab) utilisés dans différents supports de prévention des pays africains (Fig. 10). Les fabricants et les bailleurs n'ont pas suivi cette proposition.

## Enseignements et conclusion

Cette idée d'utiliser le pagne comme vecteur de sensibilisation a été peu suivie par les bailleurs, hors le pagne « *entre nous* » grâce à la ténacité de F. Deniaud.

**Figure 11:**
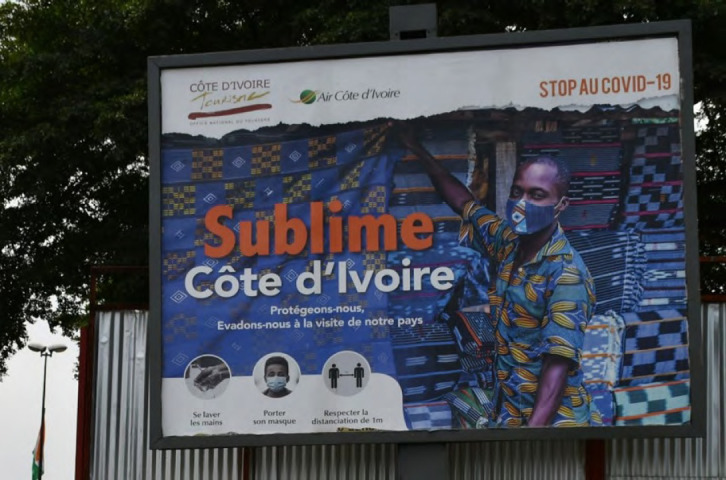
Affiche contre la Covid-19 à Abidjan en 2020 (crédit photo : Jean-Loup Rey)

Lors de la pandémie de Covid-19, nous avons proposé d'utiliser un concept identique [8] d'utilisation du pagne. L'idée n'a pas été reprise telle quelle, mais plusieurs messages faisaient allusion aux pagnes à Abidjan (Fig. 11).

Le pagne ayant fait l'objet d'une exposition au musée du Quai Branly à Paris, un bel avenir pour lui est envisageable [7] y compris dans le domaine de la prévention en santé, mais avec des approches différentes. Car, actuellement, quasiment tout le monde possède un téléphone portable avec accès à de nombreux réseaux et une sensibilisation par les pagnes présente un intérêt relatif.

## Financement

Ce travail n'a bénéficié d'aucune source de financement.

## Contribution des auteurs

Jean-Loup Rey : réalisation du texte François Deniaud : principal acteur des informations de terrain

## Déclaration d'intérêt

Les auteurs ne rapportent aucun conflit d'intérêt.
