# Supplementary material for: Loincloths and health prevention
Source: Med Trop Sante Int. 2024 Oct 22;4(4):mtsi.v4i4.2024.578. [Article in French] doi: 10.48327/mtsi.v4i4.2024.578 (PMC11892383; doi:10.48327/mtsi.v4i4.2024.578)
Supplement: Related texts

# Research article: Loincloths and health prevention

## Introduction

African loincloths are sold in every African market (Fig. 1), and every woman in sub-Saharan Africa, even the most westernized, wears a loincloth at some point. These fabrics are part of ancient African history [6] or more recent history with wax [5].

**Figure 1:** Market in Ivory Coast (photo credit: Jean-Loup Rey)

In the 90s, we considered using this fashion as a means of AIDS awareness and prevention. The aim of our work is to describe the actions taken to this end in Côte d’Ivoire, mainly in Abidjan, the economic capital. At that time, cell phones were rare, Internet use was sporadic and unaffordable.

## Preparation and use of loincloths

Three actions were carried out.

1. In 1993, the sociologist Corinne Ginoux-Pouyaud [4] interviewed the buyers of the 32 best-selling loincloths in 5 markets in Abidjan, based on information provided by the merchants. The colors and patterns of these loincloths were recorded, along with the names given by sellers and buyers. These names, which differ from those given by the manufacturers, reflect the appropriation of the loincloth by women and sometimes by men.
2. In 1993, François Deniaud [3], a medical sociologist, conducted several surveys and interviews with a hundred high school students in Abidjan and Dabou on the use of condoms and their ideas for appropriate prevention.
3. In 2001, the Women's AIDS Committee (COFEL) conducted a new survey of 50 loincloth sellers in Abidjan.

In the first survey in 1993, the dominant colors of the 32 loincloths analyzed were yellow and red (18 times), followed by blue and white. The designs were mostly abstract (scrolls, spirals, twists, grids, stripes, crosses). In 25% of the cases we found designs based on old loincloth motifs, chevrons, crosshatching and ink jets.

In terms of the names given by the women, the general themes identified are male/female relationships (25 times), happiness (19 times), beauty (11 times), money (10 times), and health (3 times). The subjects directly evoked within these themes are jealousy (5 times), wealth (5 times), love relationships (4 times), beauty (4 times), the couple, health, and ingratitude (2 times each).

Some images give a direct meaning to the name, such as the loincloth with a cage and two birds called *“tu sors, je sors” [you go, I* go] (Fig. 2), or the one (Fig. 3) with two feet on a yellow background and two on a red background called *“ton pied, mon pied” [your walk, my walk]* (where the man goes, the woman goes). A third loincloth with two horses, nicknamed *“je cours plus vite que ma rivale”* [I *run faster than my rival]* (Fig. 4), has been reissued with the previous two for several years. Geometrically patterned loincloths are called *“carrefour du bonheur” [Happiness Crossroads]* or *“attends-moi au carrefour” [wait for me at the crossroads]* (Fig. 5). More or less organic motifs are called *“œil de ma rivale” [my rival's eye]* (Fig. 6) or “la *pluie ne mouille pas le corps d'une belle fille” [Rain does not wet the body of a beautiful girl*].

**Figure 2:** Loincloth *“you go, I go”* (photo credit: Maurice Ascani)

**Figure 3:** Loincloth *“your walk, my walk”* (photo credit: Maurice Ascani)

**Figure 4:** Loincloth “I *run faster than my rival”* (photo credit: Maurice Ascani)

**Figure 5:** Loincloth *“Happiness Crossroads”* (photo credit: Jean-Loup Rey)

**Figure 6:** Loincloth *“my rival's eye”* (photo credit: Maurice Ascani)

Finally, there are the more popular event loincloths, printed on the occasion of independence or presidential anniversaries, or to mark the visit of a foreign head of state. These are not always very successful. This was the case of a loincloth - nicknamed “AIDS” - made of red amoeboid designs on a yellow background, released in 1987, when the AIDS epidemic was officially recognized in Côte d’Ivoire by the Minister of Health (Fig. 7). Similarly, the loincloth printed to commemorate the death of President Houphouët Boigny was a failure, according to the saleswomen interviewed by COFEL.

**Figure 7:** Loincloth “AIDS” placed on the market in 1987 when AIDS was officially announced (photo credit: Jean-Loup Rey)

Some loincloths have been around since the 70s, sometimes with different names: *“oeil de ma rivale” [my rival's eye]* used to be called “ *jalousie” [jealousy].* These reissues show the success of these garments, which often evoke the problems of male-female relations. In fact, these loincloths play a role in approaches to seduction or, on the contrary, rejection, as buyers are willing to play with the ambiguity of wearing a loincloth. It's not generally accepted to flirt ostentatiously with a man (especially a married man), and wearing a particular loincloth will express the unspoken: *“chéri attends-moi au carrefour” [honey, wait for me at the crossroads], “carrefour du bonheur” [Happiness Crossroads], “cœur brisé*” *[heartbroken],* but also “belle *dame”,* or a financial aspect: *“femme capable”, “mari capable”.*

All in all, a whole series of loincloths allow women to express their opinions and demands regarding their position in gender relations and against sexism: “*j'ai le droit d'aller où tu vas”* [*I have the right to go where you go*], “ *je mets ce que je veux”* [*I wear what I want*], “*l'homme est ingrat” [man is ungrateful*].

We therefore thought it would be interesting to use loincloths as a means of HIV prevention, since they are a widely used means of communication and the messages conveyed are very often related to affective or sexual relationships [4].

In 1993, a loincloth entitled *“entre nous” [between us]* was produced based on the suggestions of high school students (Fig. 8). It contains four drawings related to AIDS prevention. It was produced and distributed (Fig. 9) by François Deniaud with the help of the participating high school students and financed by the National AIDS Control Program, ORSTOM (now IRD), PSI (a social marketing company) and the French Cooperation Mission [3]. In 1995, the loincloth was included in the catalogue of information and prevention materials produced by CRIPS (AIDS Regional Information and Prevention Centre) [1]. Other ethnoprevention initiatives accompanied the marketing of the brochure: a poster designed by young people, a drawing and slogan competition, and a *“Chaussez capote” [slip on a condom]* music cassette.

**Figure 8:** Loincloth *“entre nous”* (photo credit: Jean-Loup Rey)

**Figure 9:** Loincloth *“entre nous”* introduction and distribution (photo credit: François Deniaud)

Another idea was to use an existing popular loincloth, such as the bird cage (Fig. 2), *“tu sors, je sors” [you go, I* go], with a condom for the bird in the cage, *“tu sors, je me protège” [you go, I protect myself]*, or with a condom for each bird, *“tu sors, je sors, on se protège” [you go, I go, we protect ourselves*].

In 2001, the COFEL survey involved 250 randomly selected market women, based on an interview guide on the nature of loincloths, how they are marketed, the characteristics of buyers, their intended use and the reasons for their choice.

The main results show that loincloth nicknames refer to social facts, personalities, public, traditional, or religious events, unfortunate situations, and five themes related to male-female relationships:

- Jealousy loincloths: *“œil de ma rivale” [my rival's eye], “maîtresse yako” [yako mistress]* (yako means courage in Baoule), *“ton pied, mon pied” [your walk, my walk], “chéri ne me tourne pas le dos” [honey don't turn your back on me]* or “si *tu sors, je sors” [if you go, I* go].
- Loincloths about disappointment: *“mille ans pour rien” [thousand years in vain], “si je savais” [if I* knew].
- Loincloths about the risks of sexual vagrancy: *“le monde est gâté” [the world is rotten], “bobodouman*” (the Dioula name for a sexually transmitted disease).
- Loincloths inspired by popular Brazilian television series of the time: *“Marimar”* (encounter between Marimar, a poor young girl, and a rich young man), *“sac à puces” [fleabag]* (name of Marimar's dog), *“mallette de Ricardo”, “Rosa”* and *“Matildé*” *[Ricardo's (or Rosa's, or Matildé's) briefcase],* names of characters from the TV series.
- Loinclothsabout the family: *“fleurs de mariage” [wedding flowers], “famille” [family], “gros cœur” [big heart],* “*l'enfant est mieux que l'argent” [child is better than money].*

This study confirmed the assimilation of concepts linked to certain ritual practices in the use of loincloths [2].

This work led to a proposal to create a loincloth based on the very popular *“sac à puces” [fleabag]* loincloth (named after the Brazilian TV series called Marimar), keeping the basic designs and adding AIDS symbols (umbrella, baobab) used in various prevention materials in African countries (Fig. 10). Manufacturers and donors did not follow this suggestion.

**Figure 10:** Loincloth *“sac à puces”* diverted: COFEL association proposal (photo credit: Jean-Loup Rey)

## Lessons learned and conclusions

The idea of using the loincloth as an awareness-raising tool was hardly taken up by donors, with the exception of the *“entre nous” [between us]* loincloth, thanks to the persistence of F. Deniaud. During the Covid 19 pandemic, we proposed an identical concept [8] of using the loincloth. The idea was not taken up as such, but several messages alluded to loincloths in Abidjan (Fig. 11). Now that the loincloth has been the subject of an exhibition at the Musée du Quai Branly in Paris, a bright future for it is conceivable [7], including in the field of health prevention, but with different approaches. Nowadays, almost everyone has a mobile phone with access to numerous networks, and raising awareness through loincloths is of relative interest.

**Figure 11:** Poster against Covid-19 in Abidjan in 2020 (photo credit: Jean-Loup Rey)

## Funding

No funding was provided for this work.

## Authors’ contributions

Jean-Loup Rey: writing of the text François Deniaud: main actor of the field information

## Conflict of interest

The authors declare no conflict of interests.
